# Regional cerebral pulsatile hemodynamics during isocapnic and poikilocapnic hyperthermia in young men

**DOI:** 10.14814/phy2.70258

**Published:** 2025-02-23

**Authors:** Spencer J. Skaper, Brooke R. Shepley, Ibrahim Amr Wafai, Philip N. Ainslie, Anthony R. Bain, Kurt J. Smith

**Affiliations:** ^1^ Cerebrovascular Health Exercise and Environmental Research Sciences (CHEERS) Laboratory, Department of Exercise Science, Physical & Health Education University of Victoria Victoria British Columbia Canada; ^2^ Faculty of Human Kinetics University of Windsor Windsor Ontario Canada; ^3^ Centre for Heart, Lung and Vascular Health, School of Health and Exercise Science University of British Columbia – Okanagan Kelowna British Columbia Canada

**Keywords:** carbon dioxide, cerebral blood flow, cerebral hemodynamics, hyperthermia, pulsatility

## Abstract

Hyperthermia is known to induce hypocapnia‐driven reductions in cerebral blood flow; however, it is unknown if it causes changes in hemodynamic pulsatility that negatively influence cerebrovascular function. This retrospective analysis aimed to assess cerebrovascular hemodynamic pulsatile buffering (damping factor; DFi) during poikilocapnic (HT) and isocapnic (HT‐C) hyperthermia. We hypothesized that HT would reduce cerebral DFi, while HT‐C would attenuate the reduction in DFi by limiting increases in resistance. Ten healthy males were passively heated +2°C from normothermia (BL). Blood flow through the internal carotid artery (ICA) and vertebral artery (VA) was measured using vascular ultrasound. Blood velocity through the middle cerebral artery (MCA) and the posterior cerebral artery (PCA) was measured using transcranial ultrasound. DFi was calculated as the ratio of proximal to distal pulsatility index (PI): Anterior cerebral DFi = PI_ICA_/PI_MCA_; Posterior cerebral DFi = PI_VA_/PI_PCA_. Anterior DFi decreased in both HT (1.08 ± 0.19 a.u; *p* = 0.007) and HT‐C (1.12 ± 0.231 a.u; *p* = 0.021) conditions from BL values (1.27 ± 0.14 a.u). No changes were observed in posterior DFi, *p* = 0.116. Irrespective of P_a_CO_2_ clamping, both hyperthermic conditions reduced anterior DFi, suggesting other mechanisms are responsible for cerebrovascular hemodynamic buffering. Posterior DFi responses were not observed, suggesting preferential buffering of the hyperthermic posterior circulation (VA–PCA).

## 1 INTRODUCTION

The brain is a metabolically expensive organ that demands a high blood flow relative to its size (Willie et al., 2011). However, maintaining nutrient delivery to match cerebral metabolism during environmentally challenging conditions may trigger deleterious hemodynamic patterns associated with adverse effects. For example, the thermoregulatory responses during heat stress entail profound cardiovascular and autonomic adjustments that redistribute cardiac output to the periphery (via cutaneous vasodilation) to support convective and evaporative heat loss (Crandall et al., 2008). Increased peripheral vasodilation caused by hyperthermia decreases total peripheral resistance and increases peripheral blood flow; consequently, the decreased total peripheral resistance and ensuing hypotension lower cerebral perfusion pressure [reviewed in (Bain et al., 2015)]. However, core temperature typically needs to exceed ∼1.0°C during passive hyperthermia to elicit reductions in P_a_CO_2_ between 5 and 15 mmHg (Barltrop, 1954) that result in lower CBF. Moreover, when core temperature (*T*_*c*_) increases beyond 1°C, temperature‐sensitive neurons in the medulla and carotid body cause an increase in ventilatory rate that exceeds metabolic needs, that is, hyperthermia‐induced hyperventilation (Curtis et al., 2007; Gibbons et al., 2022). The hyperthermic hyperventilation leads to a reduction in the partial pressure of arterial carbon dioxide (P_a_CO_2_; hypocapnia) causing reductions in cerebral blood flow (CBF) (Brothers et al., 2009; Nelson et al., 2011; Wilson et al., 2006). Indeed, during passive heat stress, each degree Celsius increase in *Tc* is associated with hyperventilation and an approximate 3–5 mmHg decrease in P_a_CO_2_, corresponding to an approximate 10%–15% reduction in CBF [reviewed in (Bain et al., 2015)].

Collectively, the hyperthermic‐induced hypocapnia and decreased perfusion pressure create a scenario where cerebrovascular resistances and pulsatility (PI) are altered. Typically, healthy vessels are compliant and capable of damping pulsatile hemodynamic transmittance from proximal to distal arteries despite increased cerebrovascular resistance (Muskat et al., 2021; van den Kerkhof et al., 2023; Vrselja et al., 2015; Zarrinkoob et al., 2016; Zieman et al., 2005). To date, only one study has attempted to investigate the relationship between pulsatile hemodynamic forces in central arteries and anterior cerebral blood velocities during heating (Ashton, 2013). However, this study (Zarrinkoob et al., 2016) did not measure extracranial or intracranial pulsatility to determine if hemodynamic buffering (i.e., damping; DFi) is preserved during hyperthermia and if hypocapnic vasoconstriction preserves or provokes cerebrovascular hemodynamics.

Blood flow changes affect pulsatility and the brain's buffering capacity for hemodynamic stress. For instance, decreased gCBF increases pulsatile amplitudes and reduces DFi (Dempsey et al., 2024). Because hyperthermia is known to reduce CBF, the primary aim of this retrospective analysis was to quantify the impact of hyperthermia on cerebrovascular buffering and the impact of PaCO_2_. A secondary aim was to investigate the regional cerebral DFi (i.e., anterior circulation via ICA‐MCA and posterior circulation via VA‐PCA) because of the potential for regional differences in CBF responses to heat stress (Ashton, 2013). We hypothesized that hyperthermia will reduce both posterior and anterior cerebral DFi; however, there will be no changes in DFi during isocapnic hyperthermia, suggesting that the changes in damping can be attributed to hypocapnia.

## 2 METHODS

### 2.1 Participant information

Ten healthy young males (age 23 ± 3 years) were recruited for the study. All participants were non‐obese (body mass index 23.0 ± 2.0 kg m^−1^), normotensive (sitting blood pressure 118/70 ± 6/7 mmHg), normoglycemic (<7.0 mmol l^−1^), non‐smoking, and free of overt cardiometabolic and respiratory diseases. Participants were rigorously screened to ensure optimal ICA and VA ultrasound images. Only those with ideal anatomy for sonography of the extracranial neck arteries were included (e.g., those with a high bifurcation of the common carotid artery were omitted). All experimentation was completed at the Centre for Heart, Lung & Vascular Health at the University of British Columbia, Kelowna, BC, Canada. The ethical committee of the University of British Columbia approved the study (H15‐00166). This data set was part of a larger study that focused on assessing cerebral metabolic rate of oxygen, pro‐oxidative and pro‐inflammatory markers, and endothelial‐ and platelet‐derived microparticles during passive hyperthermia. Whereas, this retrospective analysis will assess the changes to pulsatile hemodynamic forces during poikilocapnic and isocapnic passive hyperthermia. A subset of descriptive data (blood pressure [BP], heart rate [HR], temperature) from this retrospective analysis has previously been published elsewhere (Bain et al., 2017, 2020). The study conformed to the standards set by the Declaration of Helsinki, except the registry in a database. All participants provided informed written consent before experimentation.

### 2.2 Experimental protocol

After initial familiarization with the measurements, the experimental protocol was completed for each subject on a single day (between 07:00 and 18:00 h), following a 4‐h fast and a 12‐h abstinence from alcohol or caffeine. Participants were fitted into a water‐perfused tube‐lined suit (Med‐Eng, Ottawa, ON, Canada) that covered the entire body except for the head, feet, and hands. The suit was perfused with ∼49°C water until the esophageal temperature was +2°C above baseline, an absolute core temperature of 39.5°C, or the subject's volitional thermal tolerance was reached during this isolated hyperthermic exposure. Core temperature (T_*c*_) was determined by a thermocouple probe (RET‐1; Physitemp Instruments, Clifton, NJ, USA) inserted 40 cm past the nostril into the esophagus. Once peak hyperthermia was attained, the circulating temperature of the suit was reduced to ∼40°C to maintain a stable core temperature until the completion of all measures. At peak hyperthermia, P_a_CO_2_ was increased to temperature‐corrected baseline (BL) normothermic values using a custom‐built end‐tidal forcing apparatus (as explained in detail in 15). Cardiovascular and cerebrovascular measures were acquired for 1 min at BL, at +2°C during non‐randomized poikilocapnic hyperthermia (HT), and at +2°C with isocapnic hyperthermia (HT‐C).

### 2.3 Cardiovascular and cerebrovascular measures

Heart rate (HR) was obtained from the R–R intervals measured from a three‐lead ECG. Under local anesthesia (1% lidocaine) and ultrasound guidance, a 20‐gauge arterial catheter (Arrow, Markham, ON, Canada) was placed in the right radial artery, and a central venous catheter (Edwards PediaSat Oximetry Catheter, Edwards Lifesciences, Irvine, CA, USA) was placed in the right internal jugular vein and advanced towards the jugular bulb to respectively measure mean arterial pressure (MAP) and intra‐jugular venous pressure (IJVP). Heart rate and blood pressure measures were integrated into PowerLab and LabChart software (ADInstruments, Colorado Springs, CO, USA) for online monitoring and saved for offline analysis. Blood flow in the right ICA and left VA was simultaneously measured using a vascular duplex ultrasound (VDu) (Terason 3200, Teratech, Burlington, MA, USA). The right ICA was, on average, insonated 2 cm from the carotid bifurcation, while the left VA was insonated at the C5–C6 or C4–C5 space, depending on each subject's unique anatomy. The steering angle was fixed to 60 deg for all measures, and the sample volume was placed in the center of the vessel and adjusted to cover the entire vascular lumen. Additionally, blood velocity of the right middle cerebral artery (MCA) and the left posterior cerebral artery (PCA) was simultaneously measured using a transcranial Doppler (TCD) (Spencer Technologies, Seattle, WA, USA) while adhering to standardized TCD procedures (Willie et al., 2011). A specialized headband fixation device (model M600 bilateral headframe, Spencer Technologies) secured TCD probes in position throughout trials. All files were screen‐captured and saved as video files for offline analysis at 30 Hz using custom‐designed software. Simultaneous measures of luminal diameter and velocity over a minimum of 12 cardiac cycles were used to calculate blood velocity, PI, DFi, cerebrovascular resistance (CVR_i_), and conductance (CVC_i_). When a DFi is >1, it indicates greater damping of pulsatile hemodynamic forces, where a portion of the proximal arteries pulsatility is attenuated to distal arteries. A DFi equal to 1 indicates an absence of damping, allowing all the pulsatility of upstream vessels to be transmitted to downstream vessels. A DFi <1 indicates amplified pulsatility in distal arteries compared to proximal arteries. Amplification of pulsatility is hypothesized to be increased transmittance of pulsatile hemodynamic forces to the microvasculature, where chronic amplification is believed to be associated with age‐related cognitive decline (Chiesa et al., 2019; Lefferts et al., 2020). The within‐day coefficient of variation for the ICA and VA blood flow was 7% and 4%, respectively. There were no posterior velocity measures for 3 of the 10 participants.

### 2.4 Hemodynamic equations

The following hemodynamic equations were used to calculate the pulsatility index, damping factor index, MCA and PCA cerebrovascular conductance and resistance indices, ICA and VA cerebrovascular resistance, and β‐stiffness.

Pulsatility Index (PI) was calculated for the ICA, VA, MCA, and PCA as follows:

(1) $$\;{\mathrm{PI}}_{\mathrm{ICA},\mathrm{MCA},\mathrm{VA},\text{or}\;\mathrm{PCA}}\;\left(a.u\right)=\frac{\mathrm{Sys}-\text{Dias}}{\text{Velocity}}$$

where Sys is the systolic (maximum), and Dias is the diastolic (minimum) average velocities calculated for each cardiac cycle during the selected stable range. Velocity was the mean velocity over the entire chosen stable range in arbitrary units (a.u).

Anterior and posterior damping Factor index (DFi) was calculated as:

(2) $$\mathrm{DFi}\;\left(a.u\right)=\frac{{\mathrm{PI}}_{\mathrm{ICA}\;\text{or}\;\mathrm{VA}}}{{\mathrm{PI}}_{\mathrm{MCA}\;\text{or}\;\mathrm{PCA}}}$$

where PI_ICA or VA_ is the pulsatility index of the ICA or VA, and the PI_MCA or PCA_ is the pulsatility index of the MCA or PCA in arbitrary units (a.u). As previously suggested, anterior damping includes PI values from the ICA and MCA, while posterior damping includes values from the VA and PCA.

The MCA and PCA cerebrovascular conductance index (CVC_I_) was calculated as:

(3) $${\mathrm{CVC}}_{I}\;\left(\frac{\mathrm{cm}.s^{-1}}{\text{mmHg}}\right)=\frac{{\mathrm{MCA}}_{V}\;\text{or}\;{\mathrm{PCA}}_{V}}{\mathrm{MAP}}$$

where MCA_V_ or PCA_V_ is the mean velocity for the MCA or PCA, and MAP is the mean arterial pressure calculated over the cardiac cycle range.

The MCA and PCA cerebrovascular resistance index (CVR_I_), the inverse of conductance, was calculated as:

(4) $${\mathrm{CVR}}_{I}\left(\frac{\text{mmHg}}{\mathrm{cm}.s^{-1}}\right)=\frac{\mathrm{MAP}}{{\mathrm{MCA}}_{V}\;\text{or}\;{\mathrm{PCA}}_{V}}$$

where MCA_V_ or PCA_V_ is the mean velocity for the MCA or PCA, and MAP is the mean arterial pressure calculated over the cardiac cycle range.

The ICA and VA cerebrovascular resistance (CVR_ICA or VA_) was calculated as:

(5) $${\mathrm{CVR}}_{\mathrm{ICA}\;\text{or}\;\mathrm{VA}}\left(\frac{\text{mmHg}}{\mathrm{mL}.\min^{-1}}\right)=\frac{\mathrm{MAP}}{Q_{\mathrm{ICA}\;\text{or}\;\mathrm{VA}}}$$

where MAP is the mean arterial pressure calculated over Q_ICA or VA_ (internal carotid or vertebral artery blood flow).

The ICA and VA β‐stiffness was calculated using the natural logarithmic conversion of the ratio of systolic and diastolic blood pressure:

(6) $$\beta =\ln\left(\mathrm{SBP}/\mathrm{DBP}\right)\times D÷∆D$$

where ln is the natural logarithm, SBP is systolic blood pressure, DBP is diastolic blood pressure, D is diameter, and *∆*D is the difference in diameter.

### 2.5 Statistical analysis

Values are presented as mean ± standard deviation (SD). Statistical analysis was performed using JASP (Ver. 0.18.3.0, Amsterdam, the Netherlands). Within‐subject effect sizes were reported (η^2^). A repeated‐measures ANOVA was used to test for potential differences in the measured variables during hyperthermia. A Mauchly's W sphericity test was used to assess whether the assumption of sphericity was met. When sphericity was violated, a Greenhouse–Geisser correction was used to adjust the degrees of freedom. When significant differences in cerebrovascular pulsatile hemodynamic forces were detected, Holm's post hoc tests were used to determine specific differences between conditions. The a priori level of statistical significance was set at *p* < 0.05.

## 3 RESULTS

**FIGURE 1:**
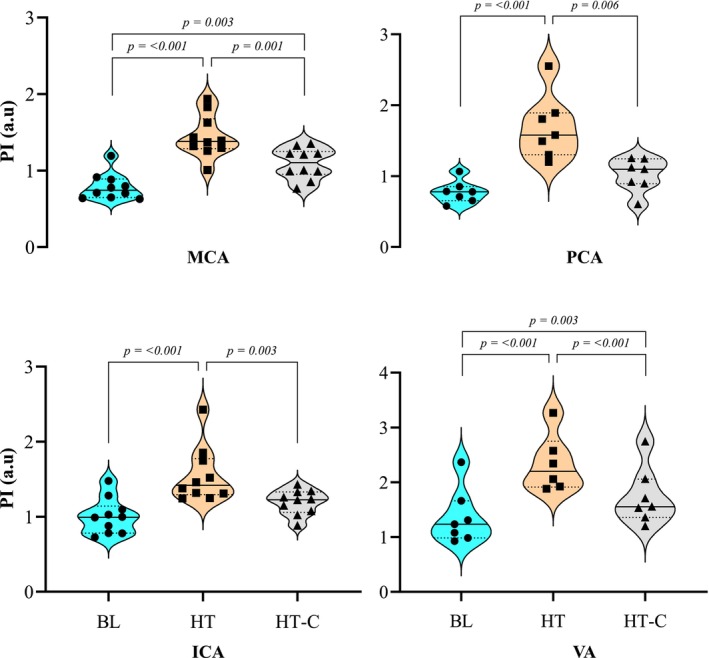
Pulsatility index (PI) (a.u) of the MCA, PCA, ICA, and VA during BL, HT, and HT‐C. Pulsatility index (PI [a.u]) of the middle cerebral (MCA), posterior cerebral (PCA), internal carotid (ICA), and vertebral (VA) arteries was demonstrated on a violin plot during baseline (BL), poikilocapnic hyperthermia (HT) and isocapnic hyperthermia (HT‐C). The black line within the violin plots indicates the group median, and the light dotted line indicates the first and third quartiles. When there were significant differences between conditions (i.e., BL vs. HT, BL vs. HT‐C, or HT vs. HT‐C) *p*‐values were presented and pairwise bars were used to illustrate where differences lie.

**FIGURE 2:**
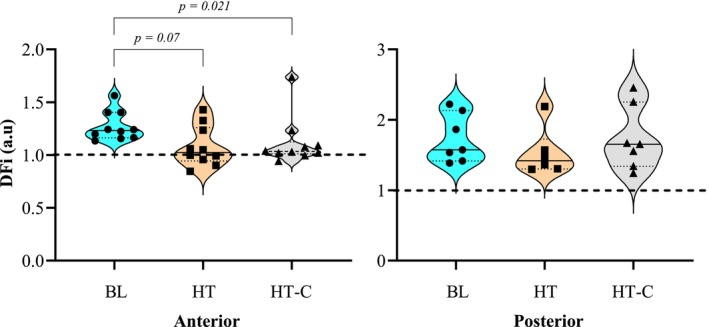
Damping factor index (DFi) (a.u) of the anterior and posterior circulation during BL, HT, and HT‐C. The damping factor index (DFi) was presented for the anterior and posterior circulation during baseline (BL), poikilocapnic hyperthermia (HT), and isocapnic hyperthermia (HT‐C). The black line within the violin plots indicates the group median, and the light dotted line indicates the first and third quartiles. Any symbols (e.g., circles, squares, and triangles) representing individual data points below the thick dashed line that goes across the entire anterior and posterior graph indicate that the individual had increased transmission of pulsatile hemodynamic forces. When there were significant differences between conditions (i.e., BL vs. HT, BL vs. HT‐C, or HT vs. HT‐C) *p*‐values were presented, and pairwise bars were used to illustrate where differences lie.

Baseline esophageal temperature was 37.1 ± 0.2°C and increased to 39.0 ± 0.4°C (HT) and 39.1 ± 0.4°C (HT‐C). MAP decreased from BL (112 ± 6.0 mmHg) during HT (96 ± 9.7 mmHg, *p* = <0.001) and HT‐C (99 ± 9.6 mmHg, *p* = <0.001). BL HR (66 ± 8 bpm) increased to 122 ± 13 bpm (HT) and 120 ± 12 bpm (HT‐C), both *p* = <0.001. Anterior (ICA and MCA) and posterior (VA and PCA) velocities, ICA and VA β‐Stiffness, CVR_MCA and PCA_, and CVC_MCA and PCA_, and IJVP are presented in Table 1. Anterior PI increased in both ICA (*η* ^2^ = 0.66) and MCA (*η* ^2^ = 0.76) from BL (1.0 ± 0.24 a.u; 0.79 ± 0.17 a.u, respectively) during HT (1.6 ± 0.4 a.u; 1.4 ± 0.28 a.u; both *p* = <0.001), but only PI_MCA_ increased from BL during HT‐C (1.1 ± 0.20 a.u, *p* = 0.003). Both ICA and MCA PI HT‐C (1.192 ± 0.165 a.u; 1.092 ± 0.201 a.u) were lower than HT (1.6 ± 0.4 a.u; 1.4 ± 0.28 a.u), *p* = 0.001. VA PI violated Mauchly's W assumption of sphericity (*p* = 0.048) and was corrected using a Greenhouse–Geisser (*p* = <0.001^a^). Posterior PI increased in both the VA (*η* ^2^ = 0.90) and PCA (*η* ^2^ = 0.70) from BL (1.388 ± 0.549 a.u; 0.775 ± 0.157 a.u, respectively) during HT (2.341 ± 0.524; 1.688 ± 0.455 a.u, *p* = <0.001), but only PI_VA_ increased from BL during HT‐C (1.797 ± 0.541 a.u; *p* = 0.003). Both HT PI_VA_ and PCA values were greater than HT‐C (1.797 ± 0.541 a.u [*p* = <0.001]; 1.018 ± 0.231 a.u [*p* = 0.006]). Anterior and posterior PI are demonstrated in Figure 1. BL Anterior DFi (1.273 ± 0.138 a.u) decreased in both HT (1.079 ± 0.19 a.u; *p* = 0.007) and HT‐C (1.117 ± 0.231 a.u; *p* = 0.021). Posterior DFi did not change, *p* = 0.116. BL CVC_MCA_ (1.827 ± 0.372 mmHg.mL.min^−1^) increased during HT (2.362 ± 0.456 mmHg.mL.min^−1^; *p* = <0.001). Anterior and posterior DFi are demonstrated in Figure 2.

**TABLE 1:** Anterior (ICA and MCA) and posterior (VA and PCA) velocity (_**V**_), ICA and VA β‐Stiffness, MCA and PCA conductances (CVC_I_) and resistances (CVR_I_), and intra jugular venous pressure (IJVP).

Variable	Cond	Mean ± stdev	*η* ^2^	Post‐hoc comparisons			Assumption checks		*p*‐Value
				BL vs. HT	BL vs. HT‐C	HT vs. HT‐C	*W*	*W p*‐value	
MCAv (cm.s)	BL	64 ± 12	0.652	<0.001**	0.253	<0.001**	0.602	0.132	<0.001**
	HT	42 ± 10							
	HT‐C	59 ± 16							
PCAv (cm.s)	BL	53 ± 14	0.716	<0.001**	0.019*	0.019*	0.605	0.222	<0.001**
	HT	35 ± 12							
	HT‐C	44 ± 18							
ICAv (cm.s)	BL	45 ± 11	0.534	0.076	0.033*	<0.001**	0.418	0.03*	0.006^a^
	HT	38 ± 9							
	HT‐C	55 ± 16							
VAv (cm.s)	BL	25 ± 10	0.366	0.364	0.365	0.115	0.809	0.654	0.103
	HT	20 ± 6							
	HT‐C	28 ± 11							
MCA CVR_I_ (mmHg.mL.min^−1^)	BL	1.83 ± 0.37	0.541	<0.001**	0.735	<0.001**	0.752	0.319	<0.001**
	HT	2.36 ± 0.46							
	HT‐C	1.79 ± 0.47							
MCA CVC_I_ (mmHg.cm.s^−1^)	BL	0.57 ± 0.11	0.613	0.005*	0.459	0.001*	0.817	0.446	<0.001**
	HT	0.44 ± 0.10							
	HT‐C	0.60 ± 0.15							
PCA CVR_I_ (mmHg.mL.min^−1^)	BL	2.24 ± 0.55	0.415	0.024*	0.324	0.116	0.765	0.448	0.024*
	HT	2.94 ± 0.84							
	HT‐C	2.47 ± 0.81							
PCA CVC_I_ (mmHg.cm.s^−1^)	BL	0.48 ± 0.15	0.414	0.032*	0.625	0.057	0.769	0.454	0.024*
	HT	0.37 ± 0.13							
	HT‐C	0.46 ± 0.21							
IJVP (mmHg)	BL	6.98 ± 3.19	0.701	0.019*	<0.001**	0.002*	0.855	0.533	<0.001**
	HT	8.29 ± 3.02							
	HT‐C	10.28 ± 3.01							
ICA β‐Stiffness	BL	9.88 ± 4.70	0.102	1.000	1.000	1.000	0.187	0.081	0.643
	HT	12.02 ± 5.62							
	HT‐C	12.06 ± 8.10							
VA β‐Stiffness	BL	19.70 ± 8.59	0.020	1.000	1.000	1.000	0.838	0.767	0.923
	HT	19.04 ± 10.74							
	HT‐C	21.00 ± 4.33							

*Note*: Mean ± standard deviation (SD) of the middle cerebral artery velocity (MCAv), posterior cerebral artery velocity (PCAv), internal carotid artery velocity (ICAv), vertebral artery velocity (VA), MCA and PCA cerebrovascular resistance (CVR_I_) and conductance (CVC_I_), intra jugular venous pressure (IJVP), and VA and ICA beta‐stiffness (β‐Stiffness) are demonstrated and post‐hoc compared during baseline (BL), poikilocapnic hyperthermic (HT), and isocapnic hyperthermic (HT‐C) conditions.

*p = <0.05 and **<0.001.

^a^ indicates a Greenhouse‐Geisser correction was used to adjust degrees of freedom when sphericity was violated. For all values, the effect size (*η* ^2^) and a Mauchly‘s W (W) sphericity test were reported.

## 4 DISCUSSION

The primary finding from this study was that poikilocapnic and isocapnic hyperthermia reduced anterior but not posterior DFi. Although hemodynamic transmittance (DFi <1) was not observed on average, 50% of subjects did incur a DFi <1 during HT, which is suggestive that there is a phenotype associated with increased transmittance. Thus, during HT, the pulsatile stress transmittance along the ICA‐MCA cerebrovascular segment is variable. Furthermore, the lack of DFi changes in the posterior circulation suggests that the posterior anatomy may limit deleterious pulsatile hemodynamic transmission from penetrating into the brain stem (Bain et al., 2015). Collectively, our results do not support our primary hypothesis, as HT and HT‐C demonstrated a reduction of DFi irrespective of P_a_CO_2_ manipulation, as shown in Figure 2. Even without a clear connection, the severity of the DFi responses was less variable in HT‐C. In addition, there was no association with arterial resistance, as MCA resistance increased during HT and did not change during HT‐C conditions. However, volumetric Q_ICA_ measures allow for a more robust measure of cerebrovascular resistance, which indicated CVR_ICA_ decreased during HT‐C (0.30 mmHg.cm.s^−1^) from BL (0.37 mmHg.cm.s^−1^) but was not altered during HT (0.37 mmHg.cm.s^−1^). Collectively, DFi responses to HT and HT‐C may require a more integrative introspection to understand the nuances associated with cerebrovascular hemodynamics during hyperthermia. Specifically, changes in intracranial pressure (ICP) and cerebral perfusion pressure, in addition to changes in vascular resistance, likely each impact hyperthermic DFi.

### 4.1 Intracranial pressure's influence on pulsatility

It is well established that hyperthermia decreases CBF but may also increase ICP (Cairns & Andrews, 2002), which is supported by recent findings using non‐invasive measures that suggest an 18% increase in ICP when moderately hyperthermic (≤39.5°C) (Gibbons et al., 2021). Mathematical models indicate that the ICP‐PI relationship is linearly related under normal conditions (Ursino & Lodi, 1997); however, further investigation is required to fully elucidate ICP‐PI interactions during hyperthermia. The IJVP (a surrogate measure for ICP) increased from BL during HT and HT‐C, with larger increases occurring when CO_2_ was clamped. IJVP is a useful surrogate for ICP in healthy volunteers as it compares well with direct measures (Cardim et al., 2019) and it is reasonable to consider that increases in CBF volume entering the cranium during HT‐C would result in an increased ICP. While speculative, our results may suggest that moderate hyperthermia may attenuate the ICP‐PI relationship. Animal models have demonstrated a >300% increase in ICP (Lin & Lin, 1992; Shih et al., 1984) during hyperthermia. Whether this increased ICP results directly from a more permeable blood–brain barrier and increased cerebral water content (~6%) (Sharma et al., 2006; Sharma, Nyberg, et al., 1992) or whether the increased ICP itself causes greater blood–brain barrier permeability and altered water content remains undetermined. Comparisons between rodent and human models are challenging, as rodents' ability to stabilize *T*_*c*_ is limited by reduced cutaneous management of hyperthermia (i.e., lack of sweating), which diminishes their capacity to represent human physiological hemodynamic responses. Increased ICP in these cases is likely associated with poor blood pressure regulation and individuals with low compliance (Nyholm et al., 2017). Other studies (Sharma et al., 2006; Sharma & Cervós‐Navarro, 1990; Sharma, Nyberg, et al., 1992; Sharma, Westman, et al., 1992) and postmortem human analysis (Hales et al., 1996; Malamud et al., 1946) have demonstrated hyperthermic cerebral edema and hemorrhaging in extreme cases. However, biomarkers indicative of blood–brain barrier disruption do not appear to be elevated in hyperthermic humans. Whether cerebral edema and hemorrhaging are influenced by increased pulsatile hemodynamic transmission remains unclear. Further investigations are needed to directly assess whether hyperthermia disrupts the ICP‐PI relationship and to elucidate its role in DFi.

### 4.2 Hyperthermic sympathetic regulation

A common perspective is that sympathetic nerve activity (SNA) vasoconstricts the cerebral vasculature (Bain et al., 2015; Brothers et al., 2009; Cassaglia et al., 2008), which would have a significant effect on cerebrovascular resistance. Indeed, passive heat stress is known to increase SNA in muscle (Low et al., 2011); however, the paucity of cerebral sympathetic‐mediated vasoconstriction and its capacity to modulate arterial function is limited (Bain et al., 2013, 2015; Nelson et al., 2011). Further, Tymko et al. (2024) indicate that blood pressure and hypercapnic challenges do not always cause cerebral sympathetic‐mediated vasoconstriction of the cerebral vasculature. Based on the understanding of central arterial compliance (i.e., arteries ability to expand and recoil throughout the cardiac cycle), acute increases in sympathetic‐adrenergic tone reduce compliance (Boutouyrie et al., 1994; Tanaka et al., 2017) and tonically restrain it in large arteries (Failla et al., 1999; Mangoni et al., 1997; Tanaka et al., 2017). Coupled with augmented forward wave intensity (Lefferts et al., 2021) and individuals' unique anatomical cerebrovascular structures (e.g., tortuous vessels and many bifurcations), the pulsatile hemodynamic transmission may increase during hyperthermia. However, it has been suggested that SNA may not cause cerebral vasoconstriction (Osol et al., 2002), and arteries distal to the Circle of Willis are unaffected (Warnert et al., 2016). Yet, changes in MAP may alter the transmural pressure and influence the depolarization of mechanosensitive cytosolic calcium ion channels that mediate resistant arteries and arterioles' myogenic reactivity, increasing wall tension (Osol et al., 2002). Thus, the calcium‐mediated myogenic reactivity in the macro and microvasculature may drive reduced DFi and DFi assessed transmission during hyperthermia. Therefore, hyperthermia may paradoxically impede the parenchymal arterioles' myogenic restrictive response, which dictates reduced cerebral perfusion pressure and prompts pulsatile hemodynamic transmission.

### 4.3 Limitations

This study was not without its experimental limitations. Intracranial measures of cerebral hemodynamics acquired using TCD are limited by the low spatial resolution offered by TCD. This limitation does not allow for MCA or PCA diameter measures; thus, assessing volumetric hemodynamics through the extra‐intracranial cerebral vessels was not possible. Studies relying on large extracranial to intracranial conduit artery hemodynamics (i.e., DFi, resistance, conductance) during thermal stimuli may benefit from including pulsatile microvascular assessments (e.g., near‐infrared spectroscopy) and mathematical approaches (i.e., modified Windkessel model) to improve our understanding of vascular compliance throughout the entire cerebrovascular tree (Moir et al., 2020; Shoemaker et al., 2023). However, it remains to be studied if a modified Windkessel model is superior to using volumetric ICA and VA resistances to understand the hemodynamic forces in the brain during hyperthermia. Furthermore, the present study had a small sample size that only included healthy males. Consequently, this limitation precluded our ability to characterize phenotypic variations or establish correlations between changes in MAP or PaCO₂ and changes in hemodynamic transmission. Indeed, future studies should include females, especially considering the paucity of studies that do not include female data desegregation. Sex hormone fluctuations over the menstrual cycle shift *T*_*c*_ and the thermoregulatory response (Kolka & Stephenson, 1997). Additionally, females tend to have a higher resting CBF compared to males (Muer et al., 2024; Rodriguez et al., 1988; Tomoto et al., 2023), although much of this CBF difference is related to lower hemoglobin in females. A lower hemoglobin content likely impacts cerebral oxygen delivery and resting state oxygen extraction fractions. For instance, the inverse relation between MCA_V_, hemoglobin content, and peak systolic velocity may indicate that females require a higher CBF to maintain adequate cerebral oxygen delivery (Mazzucco et al., 2024). However, male and female differences in resting state oxygen extraction fractions remain largely unexplored. Females do have a lower PI in comparison to their male counterparts (Alwatban et al., 2021), which may result in deleterious outcomes when exposed to hyperthermia due to their heat intolerance (Alele et al., 2020). These physiological differences underscore the need for studies investigating cerebral hemodynamics to include measures of hemoglobin content, sex hormone fluctuations, and cerebral pulsatile damping, particularly in females. Without addressing these factors, the understanding of sex‐specific responses to hyperthermia and heat stress remains incomplete, leaving a critical gap in knowledge with potentially significant implications for health and performance outcomes.

This study did not control for dehydration, which accelerates the decline in cerebral perfusion (Trangmar et al., 2015) and remains unclear during passive resting hyperthermia; however, it is likely mediated by blood pressure. Future studies should account for hydration status at a minimum and control for it as a gold standard. While direct measures of ICP were not taken due to impracticality (e.g., craniotomy), they can be obtained through non‐invasive measures (e.g., optic nerve sheath diameter measures by trans bulbar sonography (Bäuerle et al., 2013)), which would be useful to better understand the hemodynamic response to hyperthermia. In addition, future studies should consider taking bilateral measures of the VA and PCA due to their unique anatomical structure. Lastly, no measures of SNA were taken, and future studies should consider its potential neurogenic impact on cerebral arteries during hyperthermia.

## 5 CONCLUSION

The observed reductions in anterior cerebral DFi are not indicative of deleterious pulsatile hemodynamic transmission; however, there may be an individual phenotype associated with increased transmission, as 50% of individuals drop below 1.0. However, reductions in DFi and DFi transmission were only found in the anterior circulation, not the posterior. Considering the rising global temperatures, characterizing the mechanistic understanding of pulsatile hemodynamic transmission in cerebral vessels during hyperthermia can provide an important understanding of the impact of environmental temperature on brain health. Future studies need to consider both structural and functional hemodynamic regulation to better understand pulsatile hemodynamic transmission during heat stress.

## AUTHOR CONTRIBUTIONS

All authors have read and approved the final version of this manuscript and agree to be accountable for all aspects of the work to ensure that questions related to the accuracy or integrity of any part of the work are appropriately investigated and resolved. All persons designated as authors qualify for authorship, and all those who qualify are listed.

## FUNDING INFORMATION

Dr. Kurt Smith is funded through a National Sciences and Engineering Research Council (NSERC) of Canada RGPIN‐2020‐06269. Dr. Bain is funded through a National Sciences and Engineering Research Council (NSERC) of Canada RGPIN‐2020‐05760.

## CONFLICT OF INTEREST STATEMENT

None declared.

## ETHICS STATEMENT

The ethical committee of the University of British Columbia approved the study (H15‐00166). The study conformed to the standards set by the Declaration of Helsinki, except the registry database. All participants provided informed consent before experimentation.

## Data Availability

The data supporting this study's findings are available upon request. Due to proprietary restrictions, the data are not publicly available but can be accessed by qualified researchers with appropriate permissions. For inquiries regarding the data and access requests, please contact sskaper@uvic.ca.
